# How coaching leadership works: affective vs. cognitive mechanisms in Chinese internet SMEs

**DOI:** 10.3389/fpsyg.2026.1789945

**Published:** 2026-03-13

**Authors:** Hyemi Um, Juanxiu Piao, Juhee Hahn

**Affiliations:** 1 Department of Knowledge-based Management, Chung-Ang University, Seoul, Republic of Korea; 2 The Graduate School, Chung-Ang University, Seoul, Republic of Korea; 3 Department of Business Management, Chung-Ang University, Seoul, Republic of Korea

**Keywords:** coaching leadership (CL), group positive affect (GPA), incremental innovation (II), job performance (JP), psychological safety (PS)

## Abstract

**Introduction:**

This study draws upon the cognitive-affective systems theory of personality to explore the dual-mediated mechanisms linking coaching leadership to employees' incremental innovation and job performance. Specifically, we investigated the mediating roles of psychological safety (as a cognitive factor) and group positive affect (as an emotional factor) within this process.

**Methods:**

We collected data from 445 participants distributed across 92 startup teams in China and analyzed the data using multilevel analytical techniques.

**Results:**

The analysis indicated that coaching leadership is positively associated with incremental innovation and group positive affect. Furthermore, the results revealed differentiated cognitive-affective pathways: group positive affect serves as a salient affective mechanism linking coaching leadership to employee outcomes, whereas psychological safety operates in a more context-dependent manner.

**Discussion:**

These findings suggest that the influence of coaching leadership on cognitive and affective mechanisms varies across high-pressure organizational contexts. This highlights the importance of considering environmental and structural conditions when examining leadership processes to foster employee innovation and performance.

## 1 Introduction

The era of economic globalization, big data, and the mobile internet has fundamentally transformed organizational environments, placing increasing demands on leadership behaviors that can effectively mobilize and sustain employee performance (Cooper et al., 2018). In dynamic and highly competitive contexts, the effectiveness of organizational change largely depends on how leaders motivate subordinates and communicate goals, expectations, and perspectives (Liu et al., 2020). Accordingly, leadership has been widely recognized as a critical antecedent of employees' innovative behaviors and performance outcomes, attracting growing scholarly attention (Lee et al., 2020; de Haan et al., 2023).

Among various leadership approaches, coaching leadership (CL) has emerged as a particularly influential style for fostering employees' competencies and strengthening leader-follower relationships grounded in trust and mutual understanding (Alonderiene and Majauskaite, 2016; Marrone et al., 2022). By emphasizing guidance, encouragement, open communication, and empowerment, CL enhances employees' cognitive abilities and intellectual capacity while promoting effective leader-member interactions that benefit both individuals and organizations (Hagen and Gavrilova Aguilar, 2012; Ellinger, 2013; Jones et al., 2016). Prior studies have linked CL to a range of positive outcomes, including organizational citizenship behavior, job satisfaction, favorable governance perceptions in performance evaluations, and reduced turnover intentions (Moen and Federici, 2012; Özduran and Tanova, 2017; Dello Russo et al., 2017; de Haan et al., 2023).

Despite this growing body of research, the underlying mechanisms through which CL translates into employee outcomes remain insufficiently understood (Marrone et al., 2022). In particular, prior research has tended to examine the effects of CL either through cognitive or affective pathways in isolation, offering limited insight into how multiple mechanisms may operate concurrently. Drawing on the cognitive-affective systems theory of personality, individual behavior is shaped by the interaction between cognitive-affective processes and situational factors (Mischel and Shoda, 1995). This perspective suggests that leadership effects cannot be fully explained by direct relationships alone, underscoring the importance of examining mediating mechanisms that link leadership behaviors to employee outcomes (Marrone et al., 2022).

Although both cognitive and affective mechanisms have been emphasized in prior leadership research, their relative sensitivity to CL may differ across contexts characterized by high performance pressure and limited managerial discretion. In such environments-commonly observed in Internet-based small and medium-sized enterprises (SMEs)-leaders often face constrained authority and intense performance demands, which may differentially activate employees' cognitive and affective responses (Frazier et al., 2017; Edmondson and Bransby, 2023). However, existing studies have rarely specified which mechanisms are more salient under these contextual conditions, nor have they systematically compared their relative roles within a single analytical framework.

To address this gap, the present study examines how CL influences employees' incremental innovation (II) and job performance (JP) through cognitive and affective mediating mechanisms in Chinese Internet SMEs. Specifically, psychological safety (PS) is conceptualized as a cognitive mechanism, while group positive affect (GPA) is conceptualized as an affective mechanism, enabling an examination of how these mechanisms operate within the leadership process. By explicitly distinguishing and empirically comparing these two mechanisms, this study aims to clarify how CL exerts differentiated effects on employee outcomes in high-pressure, discretion-limited organizational contexts. In addition, this study adopts a multilevel analytical approach to capture leadership effects across individual and team levels.

## 2 Theoretical background and hypotheses development

### 2.1 CL and JP

According to (Whitmore 2010), coaching enables individuals to express their latent potential to achieve optimal performance. coaching leadership (CL) emphasizes an equal partnership between leaders and followers, articulates direction through vision, and prioritizes the long-term growth and development of members. Consequently, CL is considered particularly effective when managers seek to support employees in developing enduring personal strengths rather than focusing solely on short-term performance outcomes (Berg and Karlsen, 2016).

To perform the role of a coaching leader effectively, managers are required to possess a “coaching mindset,” along with the skills and abilities necessary to enact coaching behaviors in daily interactions with subordinates (Hunt and Weintraub, 2002). (Ellinger and Bostrom 2002) further argue that effective CL is characterized by trust in and empathy toward others, a reduced reliance on direct control, a willingness to support others' development, a belief in individuals' intrinsic motivation to learn, and openness to feedback. These characteristics suggest that CL fosters a supportive environment in which employees are encouraged to reflect on their work, experiment with new approaches, and continuously improve their capabilities (Marrone et al., 2022).

Employee performance refers to behaviors and actions that contribute to the attainment of organizational goals (Campbell, 1990), and performance is generally considered satisfactory when employees successfully fulfill the responsibilities associated with their roles (Campbell et al., 1993). Similar to how leaders' coaching skills help employees explore and adopt more effective ways of working and behaving, managers' coaching behaviors have been shown to enhance employee performance at both individual and group levels (Wakefield, 2006). In particular, providing constructive feedback is a core element of coaching leadership, as it facilitates learning processes and contributes directly to improvements in job performance (JP) (Mulder and Ellinger, 2013). Moreover, CL provides encouragement, guidance, authorization, and access to resources that help employees achieve their work-related objectives (Hagen, 2012; de Haan et al., 2023).

Therefore, prior research suggests that CL creates conditions that enable employees to perform their jobs more effectively by fostering learning, enhancing cognitive capabilities, and supporting goal attainment.

Thus, based on the above studies, we propose the following hypothesis:

H1: CL has a positive effect on employee's JP.

### 2.2 Mediating role of the GPA between CL and II

In increasingly complex and hyper-competitive information-oriented business contexts, innovative behavior is essential for organizational survival, sustainable success, and competitive advantage (Bagheri et al., 2022). Thus, innovative abilities or tendencies are regarded as one of the most crucial skills that business employers need (Bos-Nehles et al., 2017). (Messmann and Mulder 2012) characterized innovative behavior as a sequence of physical and cognitive actions conducted by employees, either individually or collaboratively, to accomplish tasks essential for advancing innovation.

Researchers have distinguished radical and incremental innovation (II) based on product change and novelty (Nord and Tucker, 1987). Radical innovation refers to drastic changes that are fundamentally different from existing technologies, whereas II refers to a small range of changes that appear on the continuum of current technologies (Ettlie et al., 1984). On the one hand, exploratory activities that seek new growth opportunities outside the framework of existing customers and knowledge drive radical innovation. On the other hand, activities that utilize existing skills and knowledge to meet current customer needs drive II (Benner and Tushman, 2003). Because II does not require implementing entirely new technologies and methods, it is particularly suitable for resource-constrained SMEs (Woschke and Haase, 2016).

Previous studies indicate that group positive affect (GPA) precedes desirable individual and team performance (Kelly and Barsade, 2001). By promoting new ideas and activities, GPA may elicit preferable team connections and extend attention to environmental circumstances, thoughts, and actions in the workplace (Vacharkulksemsuk and Fredrickson, 2013). Furthermore, GPA promotes team reflexivity, which enhances adaptability and collective problem-solving (Messmann and Mulder, 2015; Chi, 2022; Huang et al., 2022).

According to the broaden-and-build theory of positive emotions (Fredrickson, 2001), positive affect expands individuals' cognitive and behavioral repertoires, enabling greater flexibility and creativity. Leaders play a key role in shaping shared emotional experiences within teams, thereby influencing collective affective states (Sánchez-Cardona et al., 2018). Accordingly, CL, which emphasizes encouragement, support, and open communication, is likely to foster GPA among team members (Marrone et al., 2022).

Thus, based on the several studies we reviewed, we propose the following hypotheses:

H2: CL positively impacts the GPA.

Building on affective perspectives on innovation, we further argue that GPA enhances employees' engagement in II by promoting flexibility, reflexivity, and adaptive responses to work challenges (Chi, 2022; Huang et al., 2022).

H3: The GPA positively impacts II.

Taken together, these arguments suggest that GPA serves as an affective mechanism linking CL to employees' II.

H3-1: The GPA mediates between CL and II.

### 2.3 Mediating role of PS between CL and II

Psychological safety (PS) occurs when employees feel safe taking interpersonal risks in the workplace (Edmondson et al., 2004). According to Kahn (1990), PS enables employees to express themselves without fear of negative consequences. Prior research has identified PS as a critical cognitive condition that facilitates employee learning, voice behavior, and innovation-related activities (Choi, 2007; Cheng et al., 2008; Frazier et al., 2017).

CL represents a supportive leadership style in which managers encourage employee development and provide resources and assistance (Hagen, 2012). When employees perceive their leaders as supportive and trustworthy, they are more likely to experience PS (Mayer and Salovey, 1997). Gregory and Levy (2010) further emphasize that high-quality CL is characterized by reassurance, openness, and effective communication, all of which contribute to psychologically safe work environments (Edmondson and Bransby, 2023).

Drawing on social cognitive theory, employees' behaviors are shaped by environmental cues that influence their cognitive evaluations (Rauniyar et al., 2017). From this perspective, CL can be viewed as an important situational factor that reduces perceived risks associated with experimentation and failure, thereby enhancing PS (Frazier et al., 2017).

Thus, we propose the following hypothesis:

H4: CL positively affects PS.

Employees engaging in incremental innovation often face uncertainty and the possibility of failure (Javed et al., 2017). PS enables employees to tolerate such risks and persist in innovation-related efforts (Edmondson and Lei, 2014). Accordingly, PS is expected to facilitate employees' engagement in II (van Zyl et al., 2021).

H5: PS has a positive effect on II.

Taken together, these arguments suggest that PS functions as a cognitive mechanism through which CL influences II.

H5-1: PS mediates between CL and II.

### 2.4 II and JP

Innovative behavior entails producing and applying new ideas through experimentation (Utsch et al., 1999). Through such behavior, employees gain access to diverse information and develop deeper understandings of problems and solutions (van Zyl et al., 2021). Trial-and-error learning further expands employees' knowledge bases and enhances their capabilities (Kollmann et al., 2017).

Accordingly, II is expected to contribute positively to JP by facilitating learning and capability development (van Zyl et al., 2021).

Based on the following hypotheses, we propose that employee II positively influences JP:

H6: II positively affects employee JP.
H6-1: II mediates between GPA and employee JP.
H6-2: II mediates between PS and employee JP.

**Figure 1:**
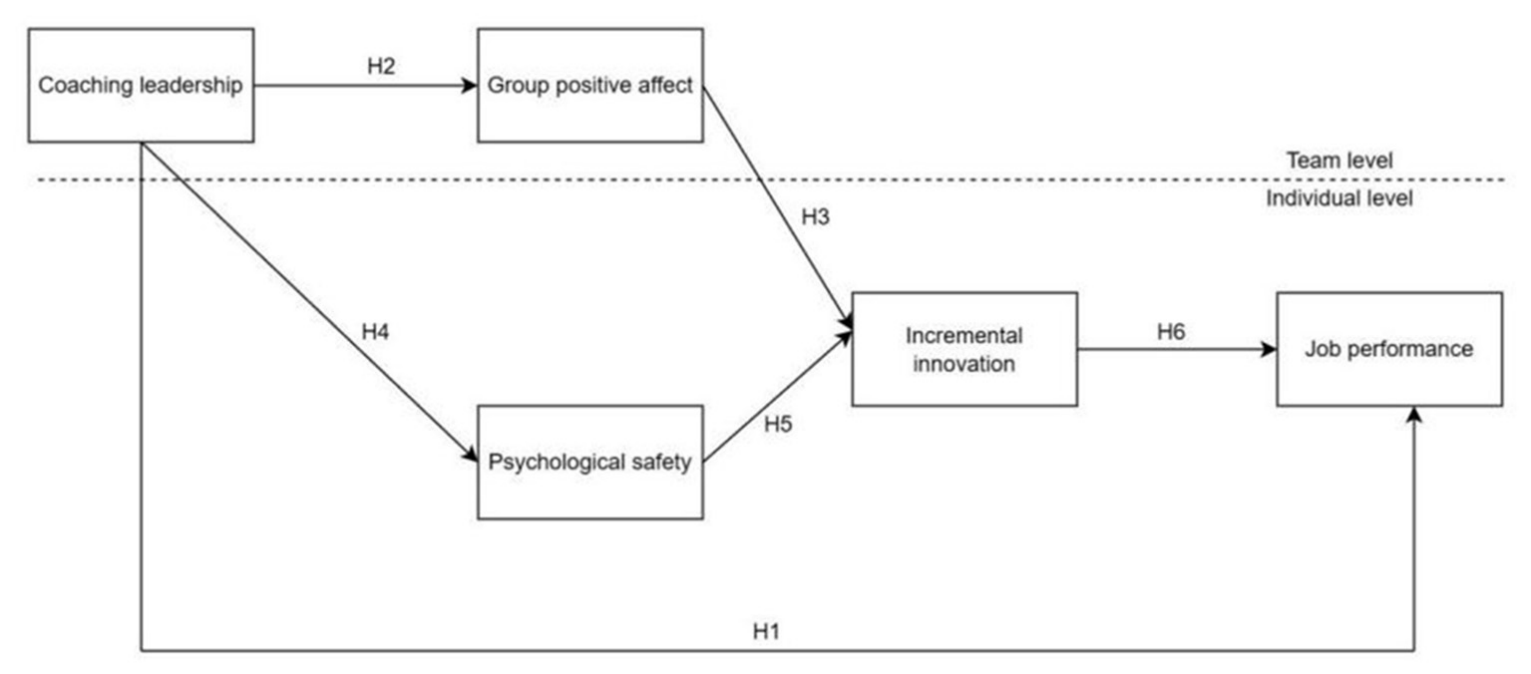
Theoretical model.

Figure 1 illustrates this study's theoretical model.

## 3 Methods

### 3.1 Participants

We collected data from 92 startup small business teams in China to examine the relationship between CL and JP. To enhance methodological rigor and minimize common method bias (CMB), we adopted a multi-wave and multi-source research design. Prior to data collection, we contacted human resource managers and team leaders of the participating firms to explain the academic purpose of the study. Participation was voluntary. To ensure data accuracy and confidentiality, a unique identification code was assigned to each leader-subordinate dyad before questionnaire distribution. This coding system allowed us to match employee responses with leader evaluations across different survey waves without revealing personal identities. Questionnaires were distributed via individualized online survey links. Participants were assured that their responses would remain anonymous and be used solely for academic research purposes. This procedure was particularly important in reducing social desirability bias when responding to sensitive constructs such as psychological safety. To further ensure data quality, we applied strict screening criteria. Questionnaires were excluded if they (a) contained incomplete responses, (b) exhibited straight-lining response patterns, or (c) failed the matching process across survey waves. Because the participants were all Chinese, we translated the entire questionnaire from English to Chinese. We conducted four surveys from August 15, 2022, to December 30, 2022. During the first survey from August 15 to August 21, 2022, we asked employees questions about CL. During the second survey, which was conducted between August 27 and September 3, we asked employees about their GPA. Then, during the third survey, which was conducted between September 10 and September 17, employees responded to questions concerning PS and II. In the third stage, we also conducted the first leaders' evaluation of employees' JP. Finally, to receive feedback about the impact of the variables on the results, we conducted our fourth survey between December 23 and December 30, 2022, with leaders completing their second evaluation of JP.

We distributed 549 surveys online and excluded invalid data after four surveys. In total, we obtained 445 valid questionnaires from employees, accounting for 81.05% of the response rate. Additionally, we obtained 92 valid questionnaires from leaders. Of the 445 employees, 46.3% (*N* = 206) were female, while 53.7% (*N* = 239) were male. The demographic group with the most employees was those aged 30–39, accounting for 42% of the total sample (*N* = 187). The employees' education comprised 45.6% (*N* = 203) with a college degree, followed by the next highest percentage of 34.4% (*N* = 153) with a bachelor's degree.

Employees with 1 to 3 years of work experience accounted for the largest proportion, that is, 27.9% (*N* = 124). Of the 92 leaders in our study, 67.4% (*N* = 62) were male, while 32.6% (*N* = 30) were female. Most leaders were between aged between 40 and 49, 41.3% (*N* = 38), and the largest proportion, 52.2% (*N* = 48), had a bachelor's degree. Regarding work experience, most leaders had seven or more years, 45.7% (*N* = 42). Finally, the sales/distribution/service industry accounted for the largest percentage of jobs, at 25% (*N* = 23).

### 3.2 Measures

Because all our respondents were all Chinese, we translated all measurement scales into Chinese using back translation (Brislin, 1980). We used a five-point Likert scale from 1 (strongly disagree) to 5 (strongly agree). Three Chinese graduate students who specialize in English reviewed the question items to ascertain the accuracy of the translations. Subsequently, we conducted focus group interviews with two graduate students and one professor to understand the questionnaire's suitability.

We assessed CL with a ten-item questionnaire developed by (Heslin et al. 2006), with a Cronbach's alpha coefficient of 0.921. This study used the ten-item GPA developed by Watson et al. (1988), resulting in a Cronbach's alpha coefficient of 0.930. In addition, this study used the six-item JP developed by Tseng and Fan (2011), achieving a Cronbach's alpha coefficient of 0.897. Moreover, our research used the three-item PS developed by Siemsen et al. (2009), resulting in a Cronbach's alpha coefficient of 0.788. Finally, this study used the 11-item II developed by Sheng and Chien (2016) and Scott and Bruce, 1994, with a resulting Cronbach's alpha coefficient of 0.946.

## 4 Data analysis and result

### 4.1 Analytical approach and CFAs (confirmatory factor analysis)

Using the Amos 23.0 program, we conducted a CFA to verify the convergent and discriminant validity of the variables before putting our hypotheses to the test. The results at the team level were as follows: χ^2^ = 286.299, DF = 169, χ^2^/df = 1.694, IFI = 0.975, TLI = 0.972, CFI = 0.975, and RMSEA = 0.040. The results at the individual level were: χ^2^ = 223.452, DF = 167, χ^2^/df = 1.338, IFI = 0.990, TLI = 0.988, CFI = 0.990, and RMSEA = 0.028. The average variance extracted (AVE) values varied from 0.542 to 0.617, exceeding the threshold of 0.50 (Hair et al., 2010). Similarly, all composite reliability values exceeded 0.70:0.922 (CL), 0.931 (GPA), 0.791 (PS), 0.946 (II), and 0.901 (JP) (Nunnally, 1978). Thus, all values satisfied the convergent validity requirements, and the fit was acceptable.

All standardized factor loadings were statistically significant and exceeded the recommended threshold of 0.60, supporting indicator reliability. Discriminant validity was further assessed using the Fornell-Larcker criterion. As shown in Table 1, the square roots of the AVE for each construct (presented on the diagonal) were greater than their corresponding inter-construct correlations, indicating satisfactory discriminant validity. Furthermore, variance inflation factors (VIFs) were below the critical threshold of 5, suggesting that multicollinearity was not a concern. In addition, the study information gathered at the individual and group levels originated from the same source (Podsakoff et al., 2003). There was no common technique variance, as evidenced by the first component's cumulative variation of 33.857%, which is less than 40%. Moreover, we used a test to determine whether the variables were multi collinear. According to our results, there was no collinearity issue because the variance inflation factor was less than 5.

**Table 1:** Results of correlation coefficients analyses.

**Individual-level variables**	**Mean**	**SD**	**1**	**2**	**3**	**4**	**5**	**6**	**7**
**(a) Individual-level variables**.									
1. Age	0.46	0.499							
2. Gender	1.82	0.840	0.073						
3. Education	2.53	0.806	−0.012	0.071					
4. Work experience	2.60	1.302	0.033	0.809^**^	0.063				
5. PS	3.639	0.945	0.015	−0.039	0.026	−0.006	(0.748)		
6. II	3.590	0.932	0.017	0.480	−0.013	0.075	0.097^*^	(0.785)	
7. JP	3.554	0.953	−0.110^*^	0.091	0.042	0.116^*^	0.003	0.335^**^	(0.778)
**(b) Team-level variables**.									
**Team-level variables**	**Mean**	**SD**	**1**	**2**	**3**	**4**	**5**	**6**	**7**
1. Team leader's age	0.33	0.471							
2. Team leader's gender	2.93	0.923	−0.203						
3. Team leader education	3.04	0.694	−0.044	−0.287^**^					
4. Team leader work experience	4.17	0.847	0.049	0.324^**^	−0.331^**^				
5. Team leader work industry	3.05	1.699	−0.160	−0.005	−0.058	0.047			
6. CL	3.631	0.483	−0.027	0.049	0.050	0.010	(0.736)		
7. GPA	3.311	0.546	0.26	0.055	−0.101	−0.078	0.286^**^	(0.758)	

CL, coaching leadership; PS, psychological safety; JP, job performance; II, incremental innovative behavior; GPA, group positive affect; N = 445 for individual-level data, and N = 92 for team-level data. ^*^ p < 0.05, ^**^ p < 0.01; Square root of AVE presented along the diagonal.

### 4.2 Descriptive statistics

Table 1 presents the means, standard deviations, and correlations among the study variables. Coaching leadership was positively correlated with group positive affect (*r* = 0.286, *p* < 0.01) and job performance, providing preliminary support for H1 and H2. Incremental innovation was positively associated with job performance (*r* = 0.335, *p* < 0.01), which is consistent with H6. Psychological safety was positively related to incremental innovation (*r* = 0.097, *p* < 0.05), offering initial support for H5. Although correlation analysis does not imply causality, these findings provide preliminary evidence consistent with the hypothesized relationships and justify the subsequent multilevel hypothesis testing.

### 4.3 Hypothesis testing

Before testing the hypotheses, we verified the existence of sufficient between-group variance regarding II and JP, as indicated by ICC1. For II, ICC1 was 0.232, chi-square = 225.508, *p* < 0.001, indicating that 23.2% of the total variance of II exists between teams. For JP, ICC1 was 0.556, chi-square = 658.833, *p* < 0.001, indicating that 55.6% of the total variance in JP exists between teams. Table 2 summarizes the HLM (Hierarchical Linear Modeling) results.

**Table 2:** Results of hierarchical regression analyses.

**Variables**	**JP**	**PS**	**II**	**II**	**II**	**II**	**JP**	**JP**	**JP**
	**Model 1**	**Model 2**	**Model 3**	**Model 4**	**Model 5**	**Model 6**	**Model 7**	**Model 8**	**Model 9**
**Level 1**									
Intercept	3.542^***^	3.641^***^	3.584^***^	3.584^***^	3.587^***^	3.587^***^	3.543^***^	3.544^***^	3.543^***^
Gender	−0.066	0.019	0.077	0.072	0.052	0.072	−0.086	−0.093	−0.08
Age	−0.001	−0.120	−0.051	−0.059	−0.014	−0.031	0.009	0.012	0.000
Education level	−0.012	0.043	−0.001	0.006	0.002	0.006	−0.016	−0.018	−0.014
Work experience	0.028	0.059	0.065	0.072	0.049	0.062	0.012	0.010	0.016
PS					0.095^*^	0.093^*^			−0.076^**^
II							0.216^***^	0.228^***^	0.224^***^
JP									
**Level 2**									
Gender	−0.095	−0.006	0.137	0.130	0.156	0.127	−0.115	−0.115	−0.116
Age	−0.066	0.050	0.116	0.113	0.120	0.083	−0.072	−0.069	−0.070
Education level	0.054	−0.121	0.083	0.071	0.040	0.002	0.072	0.060	0.063
Work experience	−0.140	−0.114	−0.113	−0.121	−0.134	−0.132^*^	−0.189	−0.116	−0.116^*^
Work industry	−0.049	−0.007	0.020	0.005	0.011	−0.023	−0.030	−0.036	−0.030
CL	0.409^***^	0.001		0.306^***^		0.680^***^			
GPA			0.350^***^	0.307^***^				−0.116	
R	0.321	0.896	0.067	0.671	0.661	0.662	0.296	0.097	0.288
Tau	0.089	0.002	0.135	0.110	0.204	0.094	0.103	0.101	0.112
Chi-square	197.257^***^	81.008	167.080^***^	144.100^***^	213.979^***^	142.138^***^	228.092^***^	222.410^***^	245.024^***^
Deviance	870.037	1245.608	1177.016	1166.007	1191.610	1158.918	848.915	848.458	845.778

CL, coaching leadership; PS, psychological safety; JP, job performance; II, incremental innovation; GPA, group positive affect. ^*^Indicates *p* < 0.05, ^**^indicates *p* < 0.01, and ^***^indicates *p* < 0.001.

Hypothesis 1 stated a positive correlation between CL and JP. As shown in Model 1 of Table 2, CL is significantly related to JP (β =0.409, *p* < 0.001), thus supporting H1. Hypothesis 4 claimed a positive relationship between CL and PS. However, Model 2 of Table 2 establishes no significant relationship between CL and PS (β =0.001, *p* > 0.05), thus rejecting H4. Hypothesis 3 stated that GPA is positively related to II. As shown in Model 3 of Table 2, GPA is significantly correlated with II (β =0.350, *p* < 0.001), thus supporting H3. Further, Hypothesis 3-1 indicated that GPA would mediate the relationship between CL and II. Model 4 in Table 2 supports this relationship between CL and II (β = 0.307, *p* < 0.001), thus supporting H3-1. Hypothesis 5 indicated a positive relationship between PS and II. Model 5 of Table 2 demonstrates a significant relationship between PS and II (β =0.095, *p* < 0.05), thus supporting H5. Next, Hypothesis 5-1 states that PS mediates between CL and II. Model 6 of Table 2 shows that PS mediates between CL and II (β =0.093, *p* < 0.05), thus supporting H5-1.

Hypothesis 6 stated that II is positively related to JP. As shown in Model 7 of Table 2, II is significantly correlated with JP (β =0.216, *p* < 0.001), thus supporting H6. In addition, Hypothesis 6-1 states that II mediates the relationship between GPA and JP. Model 8 of Table 2 shows a mediating effect between GPA and JP (β =0.228, *p* < 0.001), thus supporting H6-1. Finally, H6-2 states that II will mediate between PS and JP. Model 9 in Table 2 provides evidence of II's mediating role between PS and JP (β =0.224, *p* < 0.001), thus supporting H6-2.

Given that we assumed variables CL and GPA to be at the same level for H2, we used SPSS 26.0 for linear regression analysis. Table 3 shows that CL (β =0.286, p < 0.01) is positively related to GPA, thus supporting H2.

**Table 3:** Results of linear regression analyses.

**Variables**	**B**	**Standard error**	**β**	**t**	**p**	**Model statistics**
Intercept	2.139	0.418	–	5.118	< 0.001	R^2^ = 0.082 ΔR^2^ = 0.071
CL	0.323	0.114	0.286	2.829	0.006	*F* = 8.006, *p* = 0.006

To verify the mediating effects, we utilized multilevel structural equation modeling with the MPLUS 7 analysis method to test the robustness of our hypotheses. Table 4 shows all the direct relationships.

**Table 4:** Robustness testing: summary of mediating effects (MPLUS).

	**Estimate**	**95% CI**	**Remarks**
**Test of direct relationships**			
CL → JP	0.250^***^	(0.096, 0.403)	Supported (H1)
CL → GPA	0.389^***^	(0.172, 0.606)	Supported (H2)
GPA → II	0.656^***^	(0.483,0.828)	Supported (H3)
PS → II	0.111^**^	(0.030, 0.178)	Supported (H5)
VII → JP	0.194^***^	(0.128, 0.260)	Supported (H6)
**Test of indirect relationships**			
CL → GPA → II	0.225^**^	(0.072, 0.438)	Supported (H3-1)
GPA → II → JP	0.127^***^	(0.073, 0.181)	Supported (H6-1)
PS → II → JP	0.021^*^	(0.004, 0.038)	Supported (H6-2)

^***^p < 0.001, ^**^p < 0.01, ^*^p < 0.05.

First, we found a significant relationship between CL and JP (β = 0.250, *p* < 0.001), thus supporting Hypothesis 1. Second, because CL has a significant relationship with GPA (β = 0.389, *p* < 0.001), Hypothesis 2 is supported. Third, GPA and II (β = 0.656, *p* < 0.001) have a significant relationship, thus supporting Hypothesis 4. Fourth, we found a significant relationship between PS and II (β = 0.111, *p* < 0.01), thus supporting Hypothesis 5. Finally, we find a significant relationship between II and JP (β = 0.194, *p* < 0.001), thus supporting Hypothesis 6.

Regarding the indirect effects shown in Table 4, first, CL exhibited a significant indirect relationship with II through GPA. This relationship is indicated by the significant unstandardized estimate of the coefficient product (γ = 0.225, *p* < 0.01) and the 95% deviation-corrected CI (CI = [0.072, 0.438]) around the indirect effect; hence, supporting Hypothesis 4-1. Second, GPA has a significant indirect relationship with JP through II. This relationship is indicated by the significant unstandardized estimate of the coefficient product (γ = 0.127, *p* < 0.001) and the 95% deviation-corrected CI [CI = (0.073, 0.181)] around the indirect effect, thus supporting Hypothesis 6-1. Third, PS has a significant indirect relationship with JP through II, which is indicated by the significant unstandardized estimate of the coefficient product (γ = 0.021 *p* < 0.05) and the 95% deviation-corrected CI [CI=(0.004, 0.038)] around the indirect effect. Thus, this finding supports Hypothesis 6-2.

**Figure 2:**
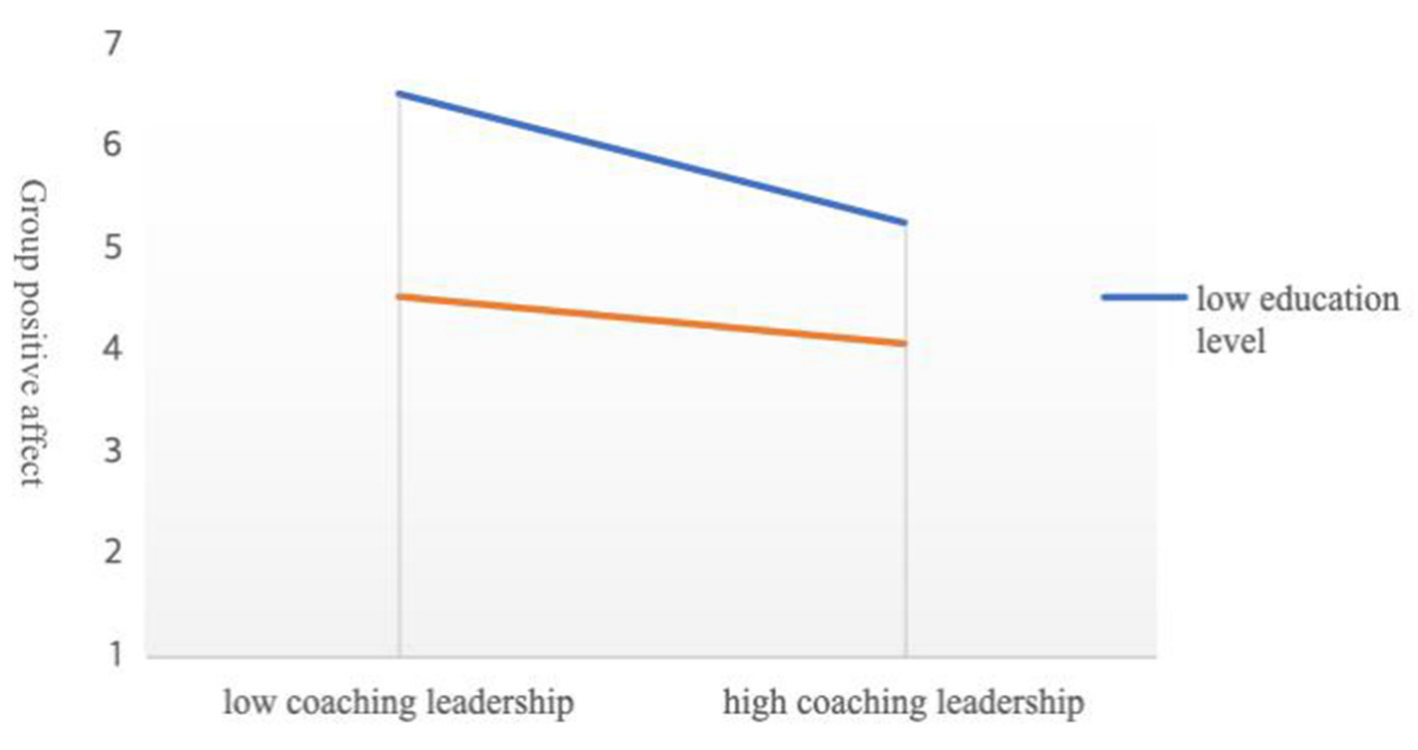
Coaching leadership and group positive affect.

Finally, we added analysis to determine whether differences in leader characteristics strengthened the relationship among the variables at the leadership levels. This study employed five variables for leader demographic characteristics: gender, age, education level, work experience, and work industry. After correlation analysis of the variables, we confirmed a significant relationship between leaders' education and leadership variables. Therefore, we divided the leaders' education level into high and low groups. As shown in Figure 2, the horizontal axis indicates the high or low level of CL, whereas the vertical axis indicates GPA. Low education level affects low CL and GPA, and the effect gradually decreases as CL increases, with a decreasing trend. Conversely, a high education level impacts low CL and GPA, and the impact gradually decreases as the CL increases, with a decreasing trend (Figure 2).

## 5 Discussion

A key finding of this study is the differentiated effect of CL on affective vs. cognitive mechanisms: while CL significantly enhances GPA, it does not exert a direct influence on PS. This distinction highlights the novelty of the present study, which moves beyond treating positive affect and PS as parallel or interchangeable outcomes of CL. Based on the cognitive-affective systems theory of personality, this study explored the dual-mediated mechanism through which CL affects incremental innovation and JP. Specifically, our findings suggest that GPA functions as an immediate affective mechanism, whereas PS operates as a downstream cognitive mechanism that facilitates the translation of innovation behaviors into performance outcomes. The study verified the direct impact of CL on employee performance and the indirect consequences through PS and GPA. We hypothesized that CL would positively impact JP, PS, and GPA and that PS and GPA mediate between CL and II. However, we obtained varied results.

We found support for H1, with CL positively impacting JP. Additionally, our findings supported H2, which argues that CL positively impacts GPA. These results are congruous with most previous studies that have demonstrated the benefits of CL (Pousa et al., 2017). In addition, this finding aligns with Ellinger et al. (2003) interpretation that a leader's coaching enhances employee performance.

However, we did not find support for H4, which claims that CL positively impacts PS. Contrary to expectations, CL did not exhibit a significant association with PS, as shown in Table 2. Indeed, some academics have indicated that this perceived safe environment has favorable results, such as increased ability, performance, and innovation (Edmondson et al., 2001). Thus, we believed establishing a positive relation between CL and PS was feasible. The unexpected findings for PS may have been caused by the following reasons.

First, we defined PS as a state in which employees are comfortable undertaking risks in the workplace (Edmondson et al., 2004), even though they may encounter many constraints executing the job in the context of innovation (Kessel et al., 2012). Our results indicate that employees perceive PS through personal judgment and relatively autonomously. Employees' evaluations of the PS level are not solely predicated on the relationship quality between them and their coaching leader (Goujon Belghit et al., 2019); organizational intention or climate also play a role. It is pertinent to note that this study's participants are exclusively small startup business members who are focused on seeking innovation and performance.

Second, the cause of our unexpected finding in the relationship between CL and PS may be the role of the coaching leader's position. Thus, we should consider the structural characteristics of team leaders. In this study, the leader is a middle manager who is leading a team of four to seven employees. Middle managers are not responsible for final decision-making but act as intermediaries between management and general employees (Harding et al., 2014). The middle manager is an essential manager and leader who leads the team and issues work instructions to subordinates. The more knowledgeable the leader, the greater their ability to enforce a stronger orientation toward corporate values (Evert et al., 2018). Implementing executives' strategic directives through top-down communication by the team leader precludes the implementation of leader coaching, which would enhance the PS of the subordinates. Because team members are usually intensely aware of their leader's conduct, leaders may unintentionally influence team members (Tyler and Lind, 1992). In addition, we discovered that GPA and PS have positive direct effects on II and mediate between CL and II. These results are consistent with previous research (Ribeiro et al., 2018). Moreover, we found that II positively impacts JP and mediates between GPA and JP. This finding indicates that influencing cognitive-emotional factors such as GPA and PS verify the mechanisms promoting JP.

Further analyzing leader characteristics, we established an interesting relationship between a leader's educational level and GPA. Low education level affects low CL and GPA, with the effect diminishing gradually and declining in magnitude as the CL increases. High education level impacts low CL and GPA; as the CL increases, the GPA tends to decrease (Figure 2). Why does a leader with a high level of education impact GPA less than a leader with a low level of education? Moreover, why does the influence on GPA decrease as CL increases in both educational levels? We noted that we conducted this study with employees of Chinese Internet SMEs. Due to the innovation process, these employees initially experience fear and anxiety; thus, they required compelling communication from leaders (Foltin and Keller, 2012).

According to the “Similarity Attraction Paradigm” (Williams and O'Reilly, 1998), people tend to attract people similar to themselves. This attraction is because they likely share common experiences or support the same values. Therefore, similar people can communicate better and work together more efficiently (Tsui and Charles A. O'reilly, 1989). In contrast, communication between dissimilar people decreases, and message distortion or communication errors increase (Williams and O'Reilly, 1998). The similarity between leaders and subordinates will increase the likelihood of being liked and satisfied, which can improve team performance by facilitating work instruction and performance (Harrison et al., 1998). However, the difference between leader and subordinates can have adverse effects, such as reducing favorability and communication (Tsui and Charles A. O'reilly, 1989). This result indicates that the influence of GPA may be influential when members have a highly homogeneous identity with their group.

### 5.1 Implications for theory and practice

This study's theoretical implications are as follows. First, by distinguishing affective and cognitive mechanisms, this study contributes to CL research by clarifying how different psychological processes operate at different stages of the innovation-performance link. Rather than conceptualizing PS as an immediate outcome of CL, our findings suggest that PS functions as a downstream cognitive mechanism that supports the conversion of II into JP. This refined view helps reconcile mixed findings in prior leadership research and extends existing models that have implicitly assumed a uniform psychological response to coaching behaviors.

This finding aligns with the arguments presented in previous literature. For example, Hsu et al. (2019) discovered that CL is a beneficial practice that guides employees, groups, and organizations to acquire new expertise, performance, and competencies for personal improvement, effectiveness, and growth. In addition, CL behaviors instill a high level of trust in the manager. Thus, employees will diligently perform their duties, enhancing overall performance.

Second, the study extends the GPA literature and research. GPA enables the exchange opinions, information, and knowledge among team members. Individual members are more likely to perceive themselves as possessing positive qualities when they attribute more positive sentiments to themselves (Buonomo et al., 2019). That is, positive feelings stimulate new and creative actions and ideas that enhance the development of physical, intellectual, social, and psychological resources, thereby enhancing the likelihood of successful responses and survival. Because individuals regulate their attitudes and behaviors through social comparisons, the workgroup's emotional climate can also significantly impact individuals (Kim, 2021). Our study confirms the stance that GPA promotes individual incremental innovation.

Third, rather than serving as an immediate outcome of CL, PS appears to function as a downstream cognitive mechanism that facilitates the translation of innovation behaviors into performance outcomes. As previously stated, CL, which is one of the main organizational situational factors, may also contribute positively to PS as a cognitive factor. In particular, CL can help decrease employees' concerns regarding the potential risks associated with risk-taking behaviors, fostering a safe, supportive, and open workplace (Anderson, 2013). Additionally, PS positively affects innovative behavior, and perceived PS helps promote innovative activities by reducing employee concerns regarding making errors (Elsayed et al., 2023).

Lastly, this study enhanced research reliability. Organizational researchers have historically focused on process and performance variables (Kluger and DeNisi, 1996). In particular, they suggested that the leader-subordinate relationship is a multilevel phenomenon that requires cross-sectional investigation and methods; however, most studies restricted themselves to a single-level analysis. Analyzing the coaching phenomenon at its level-spanning unit of analysis has become feasible because of the increasing sophistication and rigor of hierarchical analysis methods (Agarwal et al., 2009). According to this study suggests, a single-level approach may overlook critical and highly explanatory characteristics of coaching leadership-subordinate performance. The analysis in our research involved performance data on a specific individual. More precisely, we used the performance measurement data evaluated by team leaders as opposed to employee self-evaluations. However, we improved the study's reliability by reassessing the performance twice over time.

The practical implications of this study are as follows. First, we verified that CL improves individual members' II and JP. Therefore, in pursuing innovation, companies must consider CL programs as institutional investments for effective human resource management. Customized training programs that reflect the organization's characteristics and the value desired by the company will be effective. Second, management resources or specialized skills may be insufficient for SMEs. The supervisor's optimized CL will compensate for these shortcomings, resulting in rapid and reliable work results through the effective integration of new business concepts, innovative management techniques, and tools that improve the organization's vision and corporate profitability. Finally, based on this study's findings, we recommend implementing CL that reflects the environmental context, including the organization's characteristics and the value desired by the company.

### 5.2 Limitations and future research directions

This study has some limitations. First, it did not investigate the time during which the team leader managed the team members. Therefore, employees may have responded to the survey with a sense of diminished significance or utility despite the possibility that CL positively influenced them. Second, information regarding employee work structures should have been incorporated into the survey to verify more specific work behaviors and attitudes influenced by CL. Finally, additional research is required to confirm the impact of identification on the group affective process and other outcome factors. This study suggested a positive effect of PS and GPA on employee outcomes. While positive affect may hold greater significance in social interaction-oriented settings (Barsade et al., 2000), negative affect can influence a particular individual or group under particular conditions. Hence, future research should focus on the relationship between negative group affect and the behavior of group members in a specific situational context. Additionally, this study assessed the current state, refrained from investigating a more chronic condition. Therefore, future longitudinal research employing periodic measurements is required.

## 6 Conclusions

Encouraging employees to generate ideas and propose improvements is critical for organizational survival in today's highly competitive environments (Bashshur and Oc, 2015). Building on this premise, this study demonstrates that CL fosters employees' II and JP through distinct affective and cognitive pathways. The findings indicate that CL is strongly associated with GPA, which functions as a key affective mechanism linking leadership to II and JP. In contrast, PS does not emerge as a direct outcome of CL, suggesting a more context-dependent role. These results highlight that, in high-pressure Internet-based SME contexts, affective mechanisms may be more immediately responsive to CL than cognitive mechanisms. Overall, this study clarifies how CL operates through differentiated cognitive–affective pathways to promote II and JP. Future research may further examine boundary conditions—such as organizational structure, innovation stage, and environmental uncertainty—that shape the relative importance of affective and cognitive mechanisms in leadership processes.

## Data Availability

The raw data supporting the conclusions of this article will be made available by the authors, without undue reservation.
